# [2-(2-Carboxy­phen­yl)benzoato]bis­(1,10-phenanthroline)zinc(II) 2-(2-carboxy­phen­yl)benzoate monohydrate

**DOI:** 10.1107/S1600536808005862

**Published:** 2008-03-07

**Authors:** Wei-Wei Huang, Shi-Ping Yang

**Affiliations:** a School of Chemistry, Shanghai Normal University, Shanghai 200234, People’s Republic of China

## Abstract

In the title compound, [Zn(C_14_H_9_O_4_)(C_12_H_8_N_2_)_2_](C_14_H_9_O_4_)·H_2_O, the Zn^II^ atom of the complex cation is six-coordinated in an octa­hedral geometry by four N atoms from two 1,10-phenanthroline ligands and two O atoms of a carboxyl­ate group from a singly deprotonated diphenic acid. The phenanthroline and carboxylate ligands act as chelating ligands. The dihedral angles between the two benzene rings in the deprotonated diphenic acid groups are 81.05 (2) (ligand) and 89.10 (2)° (anion). O—H⋯O and C—H⋯O hydrogen bonds link the components into a three-dimensional network.

## Related literature

For related structures containing the diphenic acid anion, see: Wan & Zhang (2003 ▶); Vodak *et al.* (2001 ▶); Chui *et al.* (2001 ▶); Fernandes *et al.* (2001 ▶); Trombe *et al.* (2002 ▶); Xu *et al.* (2003 ▶); Nie *et al.* (2001 ▶); Sun *et al.* (2001 ▶).
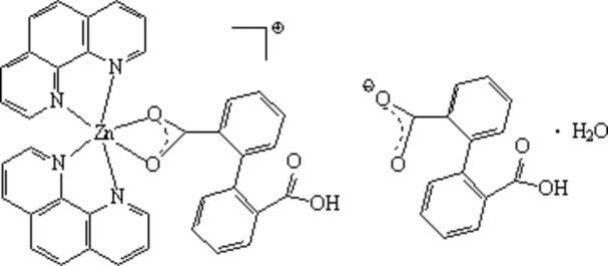


## Experimental

### 

#### Crystal data

[Zn(C_14_H_9_O_4_)(C_12_H_8_N_2_)_2_](C_14_H_9_O_4_)·H_2_O
*M* *_r_* = 926.24
Triclinic, 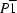

*a* = 10.8208 (11) Å
*b* = 13.7475 (14) Å
*c* = 14.9231 (16) Å
α = 77.824 (2)°
β = 77.294 (2)°
γ = 85.052 (2)°
*V* = 2115.0 (4) Å^3^
*Z* = 2
Mo *K*α radiation
μ = 0.65 mm^−1^
*T* = 298 (2) K
0.46 × 0.28 × 0.07 mm

#### Data collection

Bruker SMART CCD area-detector diffractometer
Absorption correction: multi-scan (*SADABS*; Bruker, 1998 ▶) *T*_min_ = 0.800, *T*_max_ = 0.960
13020 measured reflections
9403 independent reflections
4505 reflections with *I* > 2σ(*I*)
*R*_int_ = 0.103

#### Refinement

*R*[*F* ^2^ > 2σ(*F* ^2^)] = 0.063
*wR*(*F* ^2^) = 0.144
*S* = 0.82
9403 reflections
603 parameters
3 restraints
H atoms treated by a mixture of independent and constrained refinement
Δρ_max_ = 0.89 e Å^−3^
Δρ_min_ = −0.48 e Å^−3^

### 

Data collection: *SMART* (Bruker, 1998 ▶); cell refinement: *SAINT* (Bruker, 1998 ▶); data reduction: *SAINT*; program(s) used to solve structure: *SHELXS97* (Sheldrick, 2008 ▶); program(s) used to refine structure: *SHELXL97* (Sheldrick, 2008 ▶); molecular graphics: *SHELXTL* (Sheldrick, 2008 ▶); software used to prepare material for publication: *SHELXTL*.

## Supplementary Material

Crystal structure: contains datablocks I, global. DOI: 10.1107/S1600536808005862/ci2560sup1.cif

Structure factors: contains datablocks I. DOI: 10.1107/S1600536808005862/ci2560Isup2.hkl

Additional supplementary materials:  crystallographic information; 3D view; checkCIF report

## Figures and Tables

**Table:** 

Zn1—O2	2.078 (3)
Zn1—N1	2.128 (3)
Zn1—N4	2.130 (3)
Zn1—N3	2.145 (4)
Zn1—N2	2.145 (3)
Zn1—O1	2.334 (3)

**Table:** 

O2—Zn1—N1	102.73 (12)
O2—Zn1—N4	97.11 (12)
N1—Zn1—N4	100.12 (12)
O2—Zn1—N3	155.43 (11)
N1—Zn1—N3	101.83 (13)
N4—Zn1—N3	77.99 (14)
O2—Zn1—N2	94.88 (12)
N1—Zn1—N2	77.67 (12)
N4—Zn1—N2	167.99 (13)
N3—Zn1—N2	90.84 (13)
O2—Zn1—O1	59.17 (10)
N1—Zn1—O1	155.22 (11)
N4—Zn1—O1	98.98 (11)
N3—Zn1—O1	97.46 (11)
N2—Zn1—O1	86.71 (11)

**Table 2:** Hydrogen-bond geometry (Å, °)

*D*—H⋯*A*	*D*—H	H⋯*A*	*D*⋯*A*	*D*—H⋯*A*
O9—H9*B*⋯O7^i^	0.84 (4)	2.10 (5)	2.936 (6)	171 (6)
O4—H4*B*⋯O5^ii^	0.82	1.86	2.633 (5)	158
O9—H9*A*⋯O5	0.87 (5)	1.96 (6)	2.817 (6)	171 (7)
O8—H8*B*⋯O6	0.82	1.69	2.500 (5)	171
C3—H3⋯O1	0.93	2.55	3.130 (5)	121
C4—H4*A*⋯O6^ii^	0.93	2.42	3.221 (5)	144
C12—H12⋯O9	0.93	2.42	3.273 (6)	152
C16—H16⋯O7^i^	0.93	2.43	3.199 (7)	139
C19—H19⋯O6^iii^	0.93	2.56	3.377 (5)	146
C22—H22⋯O1^iv^	0.93	2.43	3.249 (6)	147

Symmetry codes: (i) 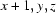; (ii) 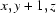; (iii) 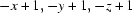; (iv) 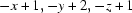.

**Fig. 1.**
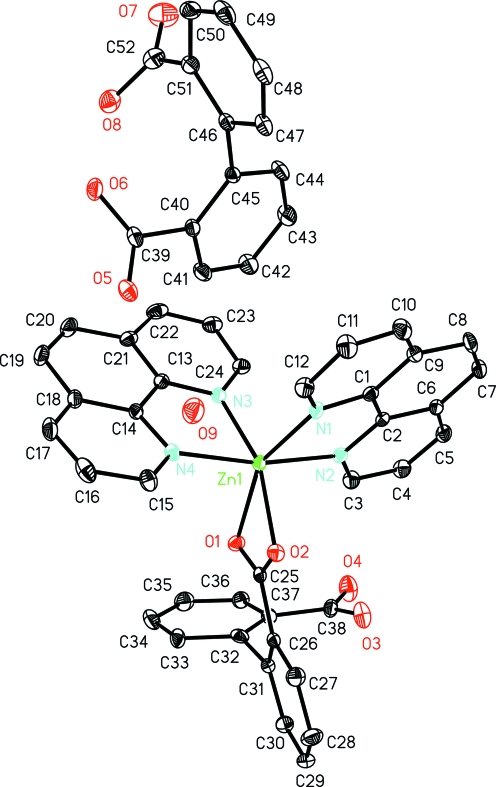
The asymmetric unit of the title compound, showing 30% probability displacement ellipsoids. H atoms have been omitted for clarity.

**Fig. 2.**
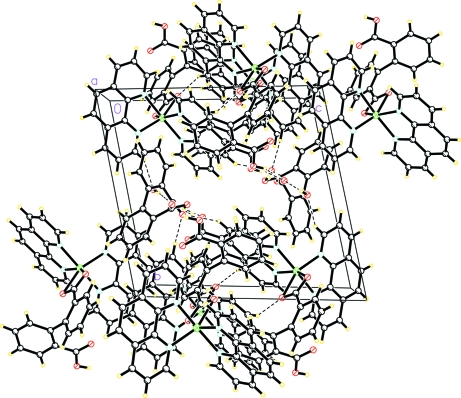
The crystal packing of the title compound, viewed down the a axis. Hydrogen bonds are shown as dashed lines.

## supplementary crystallographic information

### Comment

In all types of carboxylic acids, diphenic acid is widely used in the construction of coordination polymers due to their capability of acting as bridging and chelating ligand in various coordination modes (Wan & Zhang, 2003). Much interest has been paid to the preparation of metal aromatic carboxylates under hydrothermal conditions (Vodak *et al.*, 2001; Chui *et al.*, 2001; Nie *et al.*, 2001; Sun *et al.*, 2001; Fernandes *et al.*, 2001; Trombe *et al.* 2002; Xu *et al.* 2003). Recently, we have prepared the title compound and report here its crystal structure.

The asymmetric unit of the title compound consists of a [Zn(C_12_H_8_N_2_)_2_(C_14_H_9_O_4_)]^+^ complex cation, one singly deprotonated diphenic acid anion (C_14_H_9_O_4_^-^) and one water molecule (Fig. 1). In the complex cation, the Zn^II^ atom is six-coordinated in an octahedral geometry by four N atoms from two 1,10-phenanthroline ligands and two O atoms of a carboxylate group from a diphenic acid, with Zn—N distances of 2.128 (3)–2.145 (4) Å and Zn—O distances of 2.078 (3) and 2.334 (3) Å. The N—Zn—N, N—Zn—O and O—Zn—O bond angles are in the ranges 77.99 (14)–167.99 (13)°, 86.71 (11)–155.23 (11)° and 59.17 (10)°, respectively (Table 1). The phenanthroline and diphenic acid act as chelating ligands (Table 1 and Fig.1). The dihedral angle between the two benzene rings in diphenic acid is *ca* 81.05 (2)° (cation) and 89.10 (2)° (anion).

Intermolecular O—H···O and C—H···O hydrogen bonds (Table 2) link complex cations, anions and water molecules to form a three-dimensional network (Fig. 2).

### Experimental

A mixture of diphenic acid (0.0484 g, 0.2 mmol), phenanthroline (0.04 g, 0.2 mmol), Zn(NO_3_)_2_.6H_2_O (0.1 mmol) and NaOH (0.012 g, 0.3 mmol) with a molar ratio of 2/2/1/3 in H_2_O—CH_3_CH_2_OH (*v*/*v* 4/1) (15 ml) was sealed in a Teflon-lined stainless steel Parr bomb. The bomb was heated to 433 K for 72 h and then cooled to room temperature at 5 K h^-1^. A large amount of yellow crystals formed, which were collected by filtration, washed with water, and dried in air.

### Refinement

The water H atoms were located in a difference map and their positional parameters were refined with O—H distances restrained to 0.85 (3) Å. The remaining H atoms were positioned geometrically and refined as riding, with C—H = 0.93 Å, O—H = 0.82 Å and *U*_iso_(H) = 1.2*U*_eq_(C) and 1.2*U*_eq_(O). The components of the displacement parameters in the direction of the C31—C32 bond were restrained to be equal.

### Crystal data

**Table:**

[Zn(C_14_H_9_O_4_)(C_12_H_8_N_2_)_2_](C_14_H_9_O_4_)·H_2_O	*Z* = 2
*M**_r_* = 926.24	*F*_000_ = 956
Triclinic, *P*1	*D*_x_ = 1.454 Mg m^−^^3^
Hall symbol: -P 1	Mo *K*α radiation λ = 0.71073 Å
*a* = 10.8208 (11) Å	Cell parameters from 1630 reflections
*b* = 13.7475 (14) Å	θ = 4.8–40.2º
*c* = 14.9231 (16) Å	µ = 0.65 mm^−^^1^
α = 77.824 (2)º	*T* = 298 (2) K
β = 77.294 (2)º	Block, yellow
γ = 85.052 (2)º	0.46 × 0.28 × 0.07 mm
*V* = 2115.0 (4) Å^3^	

### Data collection

**Table:**

Bruker SMART CCD area-detector diffractometer	9403 independent reflections
Radiation source: fine-focus sealed tube	4505 reflections with *I* > 2σ(*I*)
Monochromator: graphite	*R*_int_ = 0.103
*T* = 298(2) K	θ_max_ = 28.3º
φ and ω scans	θ_min_ = 1.5º
Absorption correction: multi-scan(SADABS; Bruker, 1998)	*h* = −11→14
*T*_min_ = 0.800, *T*_max_ = 0.960	*k* = −17→17
13020 measured reflections	*l* = −16→19

### Refinement

**Table:**

Refinement on *F*^2^	Secondary atom site location: difference Fourier map
Least-squares matrix: full	Hydrogen site location: inferred from neighbouring sites
*R*[*F*^2^ > 2σ(*F*^2^)] = 0.063	H atoms treated by a mixture of independent and constrained refinement
*wR*(*F*^2^) = 0.144	*w* = 1/[σ^2^(*F*_o_^2^) + (0.0297*P*)^2^] where *P* = (*F*_o_^2^ + 2*F*_c_^2^)/3
*S* = 0.82	(Δ/σ)_max_ = 0.001
9403 reflections	Δρ_max_ = 0.89 e Å^−^^3^
603 parameters	Δρ_min_ = −0.48 e Å^−^^3^
3 restraints	Extinction correction: none
Primary atom site location: structure-invariant direct methods	

### Special details

**Table:**

Geometry. All e.s.d.'s (except the e.s.d. in the dihedral angle between two l.s. planes) are estimated using the full covariance matrix. The cell e.s.d.'s are taken into account individually in the estimation of e.s.d.'s in distances, angles and torsion angles; correlations between e.s.d.'s in cell parameters are only used when they are defined by crystal symmetry. An approximate (isotropic) treatment of cell e.s.d.'s is used for estimating e.s.d.'s involving l.s. planes.
Refinement. Refinement of *F*^2^ against ALL reflections. The weighted *R*-factor *wR* and goodness of fit *S* are based on *F*^2^, conventional *R*-factors *R* are based on *F*, with *F* set to zero for negative *F*^2^. The threshold expression of *F*^2^ > σ(*F*^2^) is used only for calculating *R*-factors(gt) *etc*. and is not relevant to the choice of reflections for refinement. *R*-factors based on *F*^2^ are statistically about twice as large as those based on *F*, and *R*- factors based on ALL data will be even larger.

### Fractional atomic coordinates and isotropic or equivalent isotropic displacement parameters (Å^2^)

**Table:**

	*x*	*y*	*z*	*U*_iso_*/*U*_eq_	
Zn1	0.64423 (5)	0.88673 (4)	0.74002 (3)	0.03550 (16)	
O1	0.7838 (3)	1.0069 (2)	0.64852 (19)	0.0426 (8)	
O2	0.8139 (3)	0.9051 (2)	0.77676 (18)	0.0405 (7)	
O3	0.7914 (4)	1.2521 (3)	0.7375 (3)	0.0767 (12)	
O4	0.7058 (4)	1.3718 (3)	0.6419 (3)	0.0865 (13)	
H4B	0.6644	1.3840	0.6916	0.130*	
N1	0.5364 (3)	0.8073 (2)	0.8672 (2)	0.0368 (9)	
N2	0.5425 (3)	1.0045 (2)	0.8031 (2)	0.0355 (8)	
N3	0.5155 (3)	0.9006 (2)	0.6470 (2)	0.0366 (9)	
N4	0.7090 (3)	0.7673 (2)	0.6686 (2)	0.0369 (9)	
C1	0.4516 (4)	0.8671 (3)	0.9161 (3)	0.0350 (10)	
C2	0.4569 (4)	0.9721 (3)	0.8828 (3)	0.0339 (10)	
C3	0.5528 (4)	1.1024 (3)	0.7720 (3)	0.0469 (12)	
H3	0.6133	1.1252	0.7185	0.056*	
C4	0.4759 (4)	1.1718 (3)	0.8172 (3)	0.0491 (12)	
H4A	0.4836	1.2396	0.7930	0.059*	
C5	0.3890 (4)	1.1393 (3)	0.8973 (3)	0.0483 (12)	
H5	0.3376	1.1850	0.9281	0.058*	
C6	0.3773 (4)	1.0374 (3)	0.9329 (3)	0.0384 (11)	
C7	0.2920 (4)	0.9964 (4)	1.0161 (3)	0.0522 (13)	
H7	0.2391	1.0390	1.0499	0.063*	
C8	0.2853 (4)	0.8969 (4)	1.0478 (3)	0.0514 (13)	
H8A	0.2276	0.8727	1.1025	0.062*	
C9	0.3653 (4)	0.8282 (3)	0.9989 (3)	0.0428 (11)	
C10	0.3674 (5)	0.7248 (4)	1.0305 (3)	0.0557 (14)	
H10	0.3105	0.6964	1.0841	0.067*	
C11	0.4531 (5)	0.6656 (4)	0.9822 (3)	0.0609 (15)	
H11	0.4568	0.5970	1.0035	0.073*	
C12	0.5357 (5)	0.7098 (3)	0.9000 (3)	0.0501 (13)	
H12	0.5927	0.6688	0.8670	0.060*	
C13	0.5415 (4)	0.8322 (3)	0.5895 (3)	0.0365 (10)	
C14	0.6448 (4)	0.7631 (3)	0.6003 (3)	0.0374 (11)	
C15	0.8014 (5)	0.6997 (3)	0.6810 (3)	0.0495 (12)	
H15	0.8443	0.7003	0.7285	0.059*	
C16	0.8374 (5)	0.6272 (3)	0.6259 (4)	0.0617 (15)	
H16	0.9042	0.5817	0.6360	0.074*	
C17	0.7750 (5)	0.6236 (4)	0.5582 (4)	0.0600 (15)	
H17	0.7984	0.5757	0.5213	0.072*	
C18	0.6766 (5)	0.6907 (3)	0.5438 (3)	0.0482 (13)	
C19	0.6023 (5)	0.6905 (4)	0.4744 (3)	0.0641 (16)	
H19	0.6232	0.6450	0.4348	0.077*	
C20	0.5024 (6)	0.7560 (4)	0.4665 (3)	0.0661 (16)	
H20	0.4551	0.7532	0.4221	0.079*	
C21	0.4670 (5)	0.8289 (4)	0.5235 (3)	0.0464 (12)	
C22	0.3630 (5)	0.8974 (4)	0.5197 (3)	0.0579 (14)	
H22	0.3120	0.8971	0.4771	0.070*	
C23	0.3367 (5)	0.9635 (4)	0.5774 (3)	0.0518 (13)	
H23	0.2673	1.0082	0.5757	0.062*	
C24	0.4157 (4)	0.9631 (3)	0.6391 (3)	0.0451 (12)	
H24	0.3976	1.0097	0.6777	0.054*	
C25	0.8448 (4)	0.9804 (3)	0.7112 (3)	0.0355 (10)	
C26	0.9559 (4)	1.0368 (3)	0.7160 (3)	0.0335 (10)	
C27	1.0267 (5)	0.9982 (4)	0.7824 (3)	0.0525 (13)	
H27	1.0055	0.9369	0.8210	0.063*	
C28	1.1267 (5)	1.0449 (4)	0.7949 (4)	0.0639 (15)	
H28	1.1724	1.0155	0.8403	0.077*	
C29	1.1583 (5)	1.1361 (4)	0.7392 (4)	0.0597 (15)	
H29	1.2254	1.1696	0.7464	0.072*	
C30	1.0889 (5)	1.1765 (4)	0.6728 (3)	0.0545 (14)	
H30	1.1105	1.2383	0.6355	0.065*	
C31	0.9879 (4)	1.1300 (3)	0.6582 (3)	0.0382 (11)	
C32	0.9304 (4)	1.1779 (3)	0.5740 (3)	0.0467 (12)	
C33	0.9827 (5)	1.1414 (3)	0.4889 (3)	0.0505 (13)	
H33	1.0407	1.0874	0.4896	0.061*	
C34	0.9473 (5)	1.1854 (4)	0.4096 (4)	0.0703 (17)	
H34	0.9760	1.1592	0.3558	0.084*	
C35	0.8636 (5)	1.2748 (4)	0.4069 (4)	0.0711 (17)	
H35	0.8431	1.3082	0.3507	0.085*	
C36	0.8155 (5)	1.3089 (4)	0.4883 (3)	0.0525 (13)	
H36	0.7614	1.3653	0.4875	0.063*	
C37	0.8477 (5)	1.2592 (4)	0.5721 (3)	0.0515 (13)	
C38	0.7835 (5)	1.2933 (4)	0.6589 (4)	0.0517 (13)	
O5	0.5364 (3)	0.4416 (3)	0.7713 (2)	0.0637 (10)	
O6	0.3732 (3)	0.3897 (2)	0.7279 (2)	0.0534 (9)	
O7	−0.0451 (4)	0.4415 (3)	0.7539 (3)	0.0927 (13)	
O8	0.1589 (4)	0.4398 (3)	0.6921 (2)	0.0643 (10)	
H8B	0.2255	0.4224	0.7097	0.096*	
C39	0.4238 (5)	0.4485 (3)	0.7635 (3)	0.0440 (12)	
C40	0.3400 (4)	0.5330 (3)	0.7956 (3)	0.0378 (11)	
C41	0.3802 (4)	0.6285 (3)	0.7610 (3)	0.0513 (13)	
H41	0.4601	0.6381	0.7225	0.062*	
C42	0.3053 (5)	0.7102 (3)	0.7819 (3)	0.0567 (14)	
H42	0.3323	0.7743	0.7553	0.068*	
C43	0.1916 (5)	0.6958 (3)	0.8416 (3)	0.0568 (14)	
H43	0.1407	0.7501	0.8577	0.068*	
C44	0.1511 (4)	0.5996 (3)	0.8790 (3)	0.0496 (13)	
H44	0.0737	0.5904	0.9211	0.059*	
C45	0.2223 (4)	0.5183 (3)	0.8554 (3)	0.0352 (10)	
C46	0.1725 (4)	0.4179 (3)	0.8974 (3)	0.0373 (11)	
C47	0.1929 (4)	0.3696 (3)	0.9849 (3)	0.0471 (12)	
H47	0.2418	0.3994	1.0149	0.057*	
C48	0.1422 (5)	0.2782 (4)	1.0288 (3)	0.0571 (14)	
H48	0.1603	0.2451	1.0859	0.069*	
C49	0.0635 (5)	0.2368 (4)	0.9855 (4)	0.0640 (16)	
H49	0.0285	0.1757	1.0142	0.077*	
C50	0.0370 (4)	0.2852 (3)	0.9012 (4)	0.0541 (14)	
H50	−0.0188	0.2583	0.8746	0.065*	
C51	0.0930 (4)	0.3744 (3)	0.8553 (3)	0.0431 (11)	
C52	0.0630 (6)	0.4223 (4)	0.7638 (4)	0.0559 (13)	
O9	0.7109 (4)	0.5128 (3)	0.8546 (3)	0.0742 (11)	
H9A	0.652 (4)	0.497 (5)	0.830 (4)	0.111*	
H9B	0.785 (3)	0.495 (5)	0.830 (4)	0.111*	

### Atomic displacement parameters (Å^2^)

**Table:**

	*U*^11^	*U*^22^	*U*^33^	*U*^12^	*U*^13^	*U*^23^
Zn1	0.0397 (3)	0.0354 (3)	0.0325 (3)	−0.0046 (2)	−0.0036 (2)	−0.0120 (2)
O1	0.043 (2)	0.0487 (19)	0.0378 (18)	−0.0073 (15)	−0.0123 (15)	−0.0061 (15)
O2	0.0471 (19)	0.0346 (17)	0.0341 (17)	−0.0083 (14)	−0.0023 (14)	0.0018 (14)
O3	0.098 (3)	0.065 (2)	0.056 (2)	0.011 (2)	0.004 (2)	−0.014 (2)
O4	0.099 (3)	0.088 (3)	0.070 (3)	0.031 (3)	−0.016 (2)	−0.026 (2)
N1	0.049 (2)	0.030 (2)	0.033 (2)	−0.0050 (17)	−0.0075 (17)	−0.0103 (16)
N2	0.039 (2)	0.033 (2)	0.036 (2)	−0.0035 (17)	−0.0071 (17)	−0.0091 (16)
N3	0.040 (2)	0.035 (2)	0.033 (2)	−0.0099 (18)	−0.0012 (17)	−0.0070 (16)
N4	0.041 (2)	0.034 (2)	0.032 (2)	−0.0026 (18)	−0.0001 (17)	−0.0033 (16)
C1	0.037 (3)	0.038 (3)	0.033 (2)	−0.005 (2)	−0.012 (2)	−0.006 (2)
C2	0.034 (3)	0.042 (3)	0.030 (2)	−0.004 (2)	−0.0107 (19)	−0.011 (2)
C3	0.057 (3)	0.037 (3)	0.044 (3)	−0.005 (2)	−0.007 (2)	−0.007 (2)
C4	0.055 (3)	0.031 (2)	0.064 (3)	0.004 (2)	−0.014 (3)	−0.015 (2)
C5	0.046 (3)	0.048 (3)	0.058 (3)	0.010 (2)	−0.013 (2)	−0.027 (3)
C6	0.036 (3)	0.046 (3)	0.036 (3)	0.000 (2)	−0.008 (2)	−0.016 (2)
C7	0.045 (3)	0.068 (4)	0.046 (3)	0.004 (3)	−0.002 (2)	−0.027 (3)
C8	0.048 (3)	0.072 (4)	0.029 (2)	0.003 (3)	0.005 (2)	−0.013 (2)
C9	0.043 (3)	0.052 (3)	0.033 (2)	−0.011 (2)	−0.002 (2)	−0.009 (2)
C10	0.066 (4)	0.053 (3)	0.039 (3)	−0.014 (3)	0.004 (2)	0.000 (2)
C11	0.082 (4)	0.040 (3)	0.050 (3)	−0.011 (3)	0.000 (3)	0.002 (2)
C12	0.060 (3)	0.042 (3)	0.046 (3)	−0.005 (3)	−0.009 (2)	−0.006 (2)
C13	0.043 (3)	0.035 (2)	0.029 (2)	−0.013 (2)	−0.002 (2)	−0.0024 (19)
C14	0.042 (3)	0.040 (3)	0.028 (2)	−0.013 (2)	0.003 (2)	−0.008 (2)
C15	0.057 (3)	0.048 (3)	0.037 (3)	0.004 (3)	−0.003 (2)	−0.003 (2)
C16	0.065 (4)	0.037 (3)	0.065 (4)	0.012 (3)	0.010 (3)	−0.003 (3)
C17	0.076 (4)	0.045 (3)	0.054 (3)	−0.009 (3)	0.014 (3)	−0.024 (3)
C18	0.056 (3)	0.046 (3)	0.041 (3)	−0.014 (3)	0.008 (2)	−0.019 (2)
C19	0.080 (4)	0.077 (4)	0.041 (3)	−0.026 (3)	0.003 (3)	−0.031 (3)
C20	0.074 (4)	0.095 (5)	0.038 (3)	−0.028 (4)	−0.009 (3)	−0.026 (3)
C21	0.053 (3)	0.055 (3)	0.031 (3)	−0.024 (3)	−0.009 (2)	0.001 (2)
C22	0.060 (4)	0.072 (4)	0.043 (3)	−0.030 (3)	−0.022 (3)	0.008 (3)
C23	0.049 (3)	0.053 (3)	0.052 (3)	−0.010 (3)	−0.016 (3)	0.002 (3)
C24	0.041 (3)	0.043 (3)	0.051 (3)	−0.007 (2)	−0.008 (2)	−0.007 (2)
C25	0.031 (3)	0.039 (3)	0.039 (3)	0.006 (2)	−0.004 (2)	−0.020 (2)
C26	0.031 (3)	0.037 (2)	0.033 (2)	0.004 (2)	−0.0042 (19)	−0.012 (2)
C27	0.054 (3)	0.055 (3)	0.047 (3)	−0.005 (3)	−0.012 (3)	−0.006 (2)
C28	0.055 (4)	0.080 (4)	0.064 (4)	−0.013 (3)	−0.026 (3)	−0.015 (3)
C29	0.038 (3)	0.084 (4)	0.067 (4)	−0.015 (3)	−0.006 (3)	−0.035 (3)
C30	0.050 (3)	0.058 (3)	0.059 (3)	−0.016 (3)	−0.003 (3)	−0.021 (3)
C31	0.032 (3)	0.046 (3)	0.038 (2)	−0.004 (2)	−0.002 (2)	−0.017 (2)
C32	0.034 (3)	0.035 (3)	0.067 (3)	−0.013 (2)	0.003 (2)	−0.012 (2)
C33	0.064 (4)	0.049 (3)	0.034 (3)	−0.013 (3)	0.005 (2)	−0.008 (2)
C34	0.056 (4)	0.098 (5)	0.051 (4)	−0.003 (3)	0.008 (3)	−0.022 (3)
C35	0.074 (4)	0.090 (5)	0.050 (3)	−0.019 (4)	−0.013 (3)	−0.007 (3)
C36	0.057 (3)	0.063 (3)	0.037 (3)	−0.014 (3)	−0.009 (2)	−0.003 (2)
C37	0.049 (3)	0.053 (3)	0.051 (3)	−0.018 (3)	0.007 (2)	−0.019 (3)
C38	0.063 (4)	0.052 (3)	0.042 (3)	−0.013 (3)	0.000 (3)	−0.019 (3)
O5	0.039 (2)	0.078 (3)	0.076 (2)	0.0133 (19)	−0.0019 (18)	−0.038 (2)
O6	0.065 (2)	0.0467 (19)	0.050 (2)	0.0005 (17)	−0.0018 (17)	−0.0241 (16)
O7	0.067 (3)	0.101 (3)	0.114 (4)	0.006 (3)	−0.042 (3)	−0.009 (3)
O8	0.076 (3)	0.073 (3)	0.048 (2)	−0.005 (2)	−0.021 (2)	−0.0099 (18)
C39	0.046 (3)	0.044 (3)	0.036 (3)	0.002 (2)	0.005 (2)	−0.009 (2)
C40	0.042 (3)	0.034 (2)	0.035 (2)	−0.003 (2)	0.001 (2)	−0.010 (2)
C41	0.047 (3)	0.048 (3)	0.052 (3)	−0.009 (3)	0.009 (2)	−0.011 (2)
C42	0.064 (4)	0.034 (3)	0.062 (3)	−0.010 (3)	0.008 (3)	−0.007 (2)
C43	0.058 (4)	0.034 (3)	0.072 (4)	0.005 (2)	0.006 (3)	−0.018 (2)
C44	0.045 (3)	0.039 (3)	0.053 (3)	0.001 (2)	0.015 (2)	−0.010 (2)
C45	0.033 (3)	0.034 (2)	0.038 (2)	−0.001 (2)	−0.002 (2)	−0.0104 (19)
C46	0.036 (3)	0.034 (2)	0.037 (3)	0.001 (2)	0.004 (2)	−0.008 (2)
C47	0.046 (3)	0.048 (3)	0.040 (3)	0.004 (2)	0.005 (2)	−0.009 (2)
C48	0.058 (4)	0.054 (3)	0.044 (3)	0.012 (3)	0.012 (3)	−0.004 (3)
C49	0.071 (4)	0.035 (3)	0.065 (4)	−0.001 (3)	0.025 (3)	−0.002 (3)
C50	0.048 (3)	0.039 (3)	0.073 (4)	−0.010 (2)	0.002 (3)	−0.018 (3)
C51	0.044 (3)	0.032 (2)	0.049 (3)	−0.002 (2)	0.000 (2)	−0.009 (2)
C52	0.060 (4)	0.045 (3)	0.066 (4)	0.002 (3)	−0.023 (3)	−0.010 (3)
O9	0.089 (3)	0.065 (3)	0.071 (3)	−0.009 (3)	−0.008 (2)	−0.024 (2)

### Geometric parameters (Å, °)

**Table:**

Zn1—O2	2.078 (3)	C23—C24	1.387 (6)
Zn1—N1	2.128 (3)	C23—H23	0.93
Zn1—N4	2.130 (3)	C24—H24	0.93
Zn1—N3	2.145 (4)	C25—C26	1.507 (5)
Zn1—N2	2.145 (3)	C26—C27	1.375 (6)
Zn1—O1	2.334 (3)	C26—C31	1.409 (6)
Zn1—C25	2.528 (4)	C27—C28	1.371 (6)
O1—C25	1.237 (5)	C27—H27	0.93
O2—C25	1.276 (5)	C28—C29	1.374 (7)
O3—C38	1.207 (5)	C28—H28	0.93
O4—C38	1.326 (6)	C29—C30	1.370 (6)
O4—H4B	0.82	C29—H29	0.93
N1—C12	1.327 (5)	C30—C31	1.391 (6)
N1—C1	1.370 (5)	C30—H30	0.93
N2—C3	1.333 (5)	C31—C32	1.525 (6)
N2—C2	1.355 (5)	C32—C37	1.369 (6)
N3—C24	1.331 (5)	C32—C33	1.446 (6)
N3—C13	1.373 (5)	C33—C34	1.331 (6)
N4—C15	1.321 (5)	C33—H33	0.93
N4—C14	1.366 (5)	C34—C35	1.462 (7)
C1—C9	1.409 (5)	C34—H34	0.93
C1—C2	1.426 (5)	C35—C36	1.374 (6)
C2—C6	1.405 (5)	C35—H35	0.93
C3—C4	1.394 (6)	C36—C37	1.394 (6)
C3—H3	0.93	C36—H36	0.93
C4—C5	1.366 (6)	C37—C38	1.478 (6)
C4—H4A	0.93	O5—C39	1.243 (5)
C5—C6	1.394 (6)	O6—C39	1.274 (5)
C5—H5	0.93	O7—C52	1.211 (6)
C6—C7	1.415 (5)	O8—C52	1.314 (6)
C7—C8	1.353 (6)	O8—H8B	0.82
C7—H7	0.93	C39—C40	1.503 (6)
C8—C9	1.430 (6)	C40—C41	1.376 (6)
C8—H8A	0.93	C40—C45	1.389 (5)
C9—C10	1.400 (6)	C41—C42	1.377 (6)
C10—C11	1.365 (6)	C41—H41	0.93
C10—H10	0.93	C42—C43	1.353 (6)
C11—C12	1.403 (6)	C42—H42	0.93
C11—H11	0.93	C43—C44	1.390 (6)
C12—H12	0.93	C43—H43	0.93
C13—C21	1.414 (6)	C44—C45	1.365 (5)
C13—C14	1.418 (6)	C44—H44	0.93
C14—C18	1.410 (6)	C45—C46	1.484 (5)
C15—C16	1.401 (6)	C46—C47	1.390 (6)
C15—H15	0.93	C46—C51	1.407 (6)
C16—C17	1.343 (7)	C47—C48	1.387 (6)
C16—H16	0.93	C47—H47	0.93
C17—C18	1.370 (7)	C48—C49	1.395 (7)
C17—H17	0.93	C48—H48	0.93
C18—C19	1.444 (7)	C49—C50	1.371 (7)
C19—C20	1.356 (7)	C49—H49	0.93
C19—H19	0.93	C50—C51	1.389 (6)
C20—C21	1.423 (6)	C50—H50	0.93
C20—H20	0.93	C51—C52	1.477 (6)
C21—C22	1.406 (7)	O9—H9A	0.87 (5)
C22—C23	1.351 (6)	O9—H9B	0.84 (2)
C22—H22	0.93		
			
O2—Zn1—N1	102.73 (12)	C23—C22—C21	120.6 (5)
O2—Zn1—N4	97.11 (12)	C23—C22—H22	119.7
N1—Zn1—N4	100.12 (12)	C21—C22—H22	119.7
O2—Zn1—N3	155.43 (11)	C22—C23—C24	118.4 (5)
N1—Zn1—N3	101.83 (13)	C22—C23—H23	120.8
N4—Zn1—N3	77.99 (14)	C24—C23—H23	120.8
O2—Zn1—N2	94.88 (12)	N3—C24—C23	124.5 (4)
N1—Zn1—N2	77.67 (12)	N3—C24—H24	117.8
N4—Zn1—N2	167.99 (13)	C23—C24—H24	117.8
N3—Zn1—N2	90.84 (13)	O1—C25—O2	121.2 (4)
O2—Zn1—O1	59.17 (10)	O1—C25—C26	122.0 (4)
N1—Zn1—O1	155.22 (11)	O2—C25—C26	116.7 (4)
N4—Zn1—O1	98.98 (11)	O1—C25—Zn1	66.7 (2)
N3—Zn1—O1	97.46 (11)	O2—C25—Zn1	55.0 (2)
N2—Zn1—O1	86.71 (11)	C26—C25—Zn1	167.7 (3)
O2—Zn1—C25	30.21 (12)	C27—C26—C31	117.9 (4)
N1—Zn1—C25	129.98 (14)	C27—C26—C25	119.0 (4)
N4—Zn1—C25	101.56 (13)	C31—C26—C25	123.1 (4)
N3—Zn1—C25	126.52 (13)	C28—C27—C26	123.7 (5)
N2—Zn1—C25	88.57 (12)	C28—C27—H27	118.1
O1—Zn1—C25	29.12 (11)	C26—C27—H27	118.1
C25—O1—Zn1	84.2 (2)	C27—C28—C29	118.9 (5)
C25—O2—Zn1	94.8 (3)	C27—C28—H28	120.6
C38—O4—H4B	109.5	C29—C28—H28	120.6
C12—N1—C1	118.0 (4)	C30—C29—C28	118.6 (5)
C12—N1—Zn1	128.5 (3)	C30—C29—H29	120.7
C1—N1—Zn1	113.3 (3)	C28—C29—H29	120.7
C3—N2—C2	118.5 (4)	C29—C30—C31	123.6 (5)
C3—N2—Zn1	127.8 (3)	C29—C30—H30	118.2
C2—N2—Zn1	113.7 (3)	C31—C30—H30	118.2
C24—N3—C13	117.3 (4)	C30—C31—C26	117.4 (4)
C24—N3—Zn1	129.6 (3)	C30—C31—C32	117.4 (4)
C13—N3—Zn1	113.1 (3)	C26—C31—C32	124.8 (4)
C15—N4—C14	117.6 (4)	C37—C32—C33	120.0 (5)
C15—N4—Zn1	129.0 (3)	C37—C32—C31	124.1 (4)
C14—N4—Zn1	113.5 (3)	C33—C32—C31	115.4 (4)
N1—C1—C9	122.2 (4)	C34—C33—C32	119.6 (5)
N1—C1—C2	117.6 (4)	C34—C33—H33	120.2
C9—C1—C2	120.1 (4)	C32—C33—H33	120.2
N2—C2—C6	122.6 (4)	C33—C34—C35	120.2 (5)
N2—C2—C1	117.1 (4)	C33—C34—H34	119.9
C6—C2—C1	120.3 (4)	C35—C34—H34	119.9
N2—C3—C4	122.2 (4)	C36—C35—C34	119.2 (5)
N2—C3—H3	118.9	C36—C35—H35	120.4
C4—C3—H3	118.9	C34—C35—H35	120.4
C5—C4—C3	119.4 (4)	C35—C36—C37	120.2 (5)
C5—C4—H4A	120.3	C35—C36—H36	119.9
C3—C4—H4A	120.3	C37—C36—H36	119.9
C4—C5—C6	120.0 (4)	C32—C37—C36	120.7 (4)
C4—C5—H5	120.0	C32—C37—C38	121.5 (5)
C6—C5—H5	120.0	C36—C37—C38	117.7 (5)
C5—C6—C2	117.3 (4)	O3—C38—O4	122.4 (5)
C5—C6—C7	124.3 (4)	O3—C38—C37	125.5 (5)
C2—C6—C7	118.4 (4)	O4—C38—C37	111.9 (5)
C8—C7—C6	121.8 (4)	C52—O8—H8B	109.5
C8—C7—H7	119.1	O5—C39—O6	124.5 (4)
C6—C7—H7	119.1	O5—C39—C40	119.0 (4)
C7—C8—C9	121.3 (4)	O6—C39—C40	116.4 (4)
C7—C8—H8A	119.3	C41—C40—C45	119.1 (4)
C9—C8—H8A	119.3	C41—C40—C39	118.3 (4)
C10—C9—C1	117.8 (4)	C45—C40—C39	122.5 (4)
C10—C9—C8	124.1 (4)	C40—C41—C42	121.8 (4)
C1—C9—C8	118.0 (4)	C40—C41—H41	119.1
C11—C10—C9	119.8 (4)	C42—C41—H41	119.1
C11—C10—H10	120.1	C43—C42—C41	118.9 (4)
C9—C10—H10	120.1	C43—C42—H42	120.5
C10—C11—C12	119.1 (5)	C41—C42—H42	120.5
C10—C11—H11	120.5	C42—C43—C44	119.9 (4)
C12—C11—H11	120.5	C42—C43—H43	120.0
N1—C12—C11	123.1 (4)	C44—C43—H43	120.0
N1—C12—H12	118.5	C45—C44—C43	121.5 (4)
C11—C12—H12	118.5	C45—C44—H44	119.2
N3—C13—C21	121.7 (4)	C43—C44—H44	119.2
N3—C13—C14	117.4 (4)	C44—C45—C40	118.6 (4)
C21—C13—C14	120.9 (4)	C44—C45—C46	118.6 (4)
N4—C14—C18	121.7 (4)	C40—C45—C46	122.8 (4)
N4—C14—C13	117.9 (4)	C47—C46—C51	118.4 (4)
C18—C14—C13	120.4 (4)	C47—C46—C45	119.9 (4)
N4—C15—C16	122.8 (5)	C51—C46—C45	121.3 (4)
N4—C15—H15	118.6	C48—C47—C46	121.7 (5)
C16—C15—H15	118.6	C48—C47—H47	119.2
C17—C16—C15	119.6 (5)	C46—C47—H47	119.2
C17—C16—H16	120.2	C47—C48—C49	118.6 (5)
C15—C16—H16	120.2	C47—C48—H48	120.7
C16—C17—C18	119.9 (5)	C49—C48—H48	120.7
C16—C17—H17	120.1	C50—C49—C48	120.8 (5)
C18—C17—H17	120.1	C50—C49—H49	119.6
C17—C18—C14	118.5 (5)	C48—C49—H49	119.6
C17—C18—C19	123.5 (5)	C49—C50—C51	120.3 (5)
C14—C18—C19	118.0 (5)	C49—C50—H50	119.8
C20—C19—C18	120.6 (5)	C51—C50—H50	119.8
C20—C19—H19	119.7	C50—C51—C46	120.0 (5)
C18—C19—H19	119.7	C50—C51—C52	118.0 (5)
C19—C20—C21	122.5 (5)	C46—C51—C52	121.9 (4)
C19—C20—H20	118.7	O7—C52—O8	121.0 (5)
C21—C20—H20	118.7	O7—C52—C51	121.9 (5)
C22—C21—C13	117.5 (4)	O8—C52—C51	117.0 (5)
C22—C21—C20	125.0 (5)	H9A—O9—H9B	115 (6)
C13—C21—C20	117.5 (5)		

### Hydrogen-bond geometry (Å, °)

**Table:**

*D*—H···*A*	*D*—H	H···*A*	*D*···*A*	*D*—H···*A*
O9—H9B···O7^i^	0.84 (4)	2.10 (5)	2.936 (6)	171 (6)
O4—H4B···O5^ii^	0.82	1.86	2.633 (5)	158
O9—H9A···O5	0.87 (5)	1.96 (6)	2.817 (6)	171 (7)
O8—H8B···O6	0.82	1.69	2.500 (5)	171
C3—H3···O1	0.93	2.55	3.130 (5)	121
C4—H4A···O6^ii^	0.93	2.42	3.221 (5)	144
C12—H12···O9	0.93	2.42	3.273 (6)	152
C16—H16···O7^i^	0.93	2.43	3.199 (7)	139
C19—H19···O6^iii^	0.93	2.56	3.377 (5)	146
C22—H22···O1^iv^	0.93	2.43	3.249 (6)	147

Symmetry codes: (i) *x*+1, *y*, *z*; (ii) *x*, *y*+1, *z*; (iii) −*x*+1, −*y*+1, −*z*+1; (iv) −*x*+1, −*y*+2, −*z*+1.
